# The Female Reproductive Tract Microbiome and Cancerogenesis: A Review Story of Bacteria, Hormones, and Disease

**DOI:** 10.3390/diagnostics13050877

**Published:** 2023-02-24

**Authors:** Oana Gabriela Trifanescu, Raluca Alexandra Trifanescu, Radu Iulian Mitrica, Diana Maria Bran, Georgia Luiza Serbanescu, Laurentiu Valcauan, Serban Andrei Marinescu, Laurentia Nicoleta Gales, Bogdan Cosmin Tanase, Rodica Maricela Anghel

**Affiliations:** 1 Department of Oncology, „Carol Davila” University of Medicine and Pharmacy, 022328 Bucharest, Romania; 2 2nd Department of Radiotherapy, „Prof. Dr. Al. Trestioreanu” Institute of Oncology, 022328 Bucharest, Romania; 3 Department of Endocrinology, “Carol Davila” University of Medicine and Pharmacy, 011863 Bucharest, Romania; 4 “C.I. Parhon” Institute of Endocrinology, 011863 Bucharest, Romania; 5 Department of Surgery, „Prof. Dr. Al. Trestioreanu” Institute of Oncology, 022328 Bucharest, Romania; 6 2nd Department of Oncology, „Prof. Dr. Al. Trestioreanu” Institute of Oncology, 022328 Bucharest, Romania; 7 Department of Thoracic Surgery, “Prof. Dr. Al. Trestioreanu” Institute of Oncology, 022328 Bucharest, Romania

**Keywords:** microbiome, carcinogenesis, female reproductive tract, immunotherapy

## Abstract

The microbiota is the complex community of microorganisms that populate a particular environment in the human body, whereas the microbiome is defined by the entire habitat—microorganisms and their environment. The most abundant and, therefore, the most studied microbiome is that of the gastrointestinal tract. However, the microbiome of the female reproductive tract is an interesting research avenue, and this article explores its role in disease development. The vagina is the reproductive organ that hosts the largest number of bacteria, with a healthy profile represented mainly by *Lactobacillus* spp. On the other hand, the female upper reproductive tract (uterus, Fallopian tubes, ovaries) contains only a very small number of bacteria. Previously considered sterile, recent studies have shown the presence of a small microbiota here, but there are still debates on whether this is a physiologic or pathologic occurrence. Of particular note is that estrogen levels significantly influence the composition of the microbiota of the female reproductive tract. More and more studies show a link between the microbiome of the female reproductive tract and the development of gynecological cancers. This article reviews some of these findings.

## 1. Introduction

### 1.1. The Microbiome of the Female Reproductive Tract

The healthy vagina harbors a microbiota characterized by a low diversity of species, represented mainly by *Lactobacillus* spp. (*Lactobacillus crispatus*, *Lactobacillus gasseri*, *Lactobacillus iners*, *Lactobacillus jensenii*, *Lactobacillus vaginalis*) [1,2,3]. This starkly contrasts with the high diversity of species demonstrated by the healthy colon [3,4,5,6]. The vagina contains species that process glycogen and its breakdown products to produce lactic acid, thus leading to an acidic pH of less than 4.5 [7]. This is important because it inactivates pathogens and prevents the ascent of pathogenic bacteria to the upper reproductive tract. Lactobacilli also secrete antimicrobial products and prevent the adhesion of pathogens [8].

On the pathological side, endometriosis, gynecological cancers and fertility problems may all be related to uterine microbiota [3].

Vaginal dysbiosis is characterized by a high diversity of bacterial species and a high pH. A diseased vaginal environment contains a mixture of anaerobic bacteria such as *Sneathia* spp., *Atopobium* spp., *Porphyromonas* spp., *Gardnerella vaginalis* etc. It can sometimes contain bacteria such as *Streptococcus* spp., *Staphylococcus* spp. and *Enterobacteriaceae*. In vaginal dysbiosis, the *Lactobacillus* spp. are low in number leading to an increased risk of bacterial vaginosis. The specific types of lactobacilli also matter. For instance, a vagina that contains mainly *Lactobacillus iners* frequently transitions to become anaerobe-dominant [2,9]. However, not all *Lactobacillus* species have the same effect; for example, this transition fails to develop in a vagina with predominant *Lactobacillus crispatus*. This might be related to the type of lactic acid produced by each bacterium. *Lactobacillus iners* only synthesizes the L-isoform of lactic acid, which correlates with higher levels of metalloproteinases in vaginal secretions and lesser epithelial integrity of the vaginal wall [2,10]. This is of practical importance in deciding the appropriate *Lactobacillus* spp. to use as vaginal probiotics.

The upper female reproductive tract (uterus, Fallopian tubes and ovaries) was considered sterile for a long time. Recent molecular studies showed that it might harbor its own microbiota, but it is unclear if the samples used in studies were not contaminated during collection [2]. The healthy upper reproductive tract would contain only a very small biomass of bacteria whose composition and implication for the woman’s and baby’s health is under investigation. This biomass’s exact composition and diversity are still scrutinized, but it would probably contain a smaller percentage of lactobacilli than the vagina.

In contrast, the diseased and pathologic upper reproductive tract often contains a large biomass. Among the bacteria that colonize the upper genital tract, some can be particularly aggressive, leading to infertility, such as *Chlamydia*, *Mycoplasma*, *Acinetobacter*, and *Brucella*. On the other hand, certain bacteria, such as *Atopobium* and *Porphyromonas*, have been shown to correlate with endometrial hyperplasia and endometrial cancer [2]. These bacteria usually colonize the upper reproductive tract by ascending from the vagina, but there may also be direct hematogenous seeding [3].

In addition to the female reproductive tract there are two other important female microbiomes, and they all influence each other. The other two microbiomes are that of the urethra and bladder, and that of the anus and rectum. The composition of each organ’s microbiota is influenced by the direct transfer of microorganisms from the other organs. Both the urethra and rectum contain *Lactobacillus* spp. [11].

### 1.2. Estrogens and the Estrobolome

In addition to the reproductive tract flora, another major component that influences the reproductive tract environment is represented by the female hormones, particularly estrogens. This part of the article explores the link between estrogens and the microbiome. 

Estrogens are conjugated in the liver by sulfotransferase and uridine diphosphate—glucuronosyltransferase enzymes and then excreted into the gut through the bile. In the gut, some conjugated estrogens are deconjugated by beta-glucuronidase and beta-glucosidase and then reabsorbed through the intestinal epithelium back into the bloodstream [2]. Interestingly, these enzymes can be produced by some gut bacteria. Thus, the gut microbiota’s composition directly impacts circulating estrogen levels [12,13].

The estrobolome is nowadays defined as the aggregate of all enteric bacterial genes whose products are capable of metabolizing estrogens [2].

The activity of different enzymes, such as β-glucuronidase, is encoded mainly by two genes. First is Gus, found in Firmicutes [14], and second is BG, found in Bacteroidetes [15]. β-glucuronidase activity is also influenced by diet [16]. A high-fat diet may increase bile acid secretion, promoting Proteobacteria growth and reducing Bacteroidetes and Firmicutes [17].

Increases in β-glucuronidase-producing Proteobacteria increase intestinal deconjugation of estrogens and estrogens levels in circulation. This mechanism is intensified in obese patients, mainly due to peripheral aromatization of testosterone and androstenedione to estradiol and estrone [18,19].

Since estrogen levels are associated with various types of cancers, such as endometrial or breast cancer, we can hypothesize that the estrobolome also impacts the carcinogenesis of these types of cancers.

Moreover, the composition of the vaginal microbiome is deeply impacted by estrogen levels. Before puberty and after menopause, the vaginal microbiome consists primarily of anaerobes, whereas for healthy females of reproductive age, the vaginal microbiome consists mainly of *Lactobacillus* spp. [8,20]. Indeed, estrogens stimulate the production and secretion of glycogen by the vaginal epithelium, promoting the growth of lactobacilli. Lactobacilli then use glycogen as a food source and degrade it through fermentation. Large amounts of the lactic acid result as a final product of this process [2,8,21].

Therefore, we can say that the vagina’s acidic environment is a direct consequence of the estrogen circulating levels. As seen above, estrogen levels are influenced, among others, by the gut microbiome.

## 2. The Microbiome and Cancer Development

Many factors promote a healthy flora versus dysbiosis, usually promoting functioning cells versus cancer. In a considerable measure, these factors are the same. In other words, the factors associated with an unhealthy gut or vaginal flora are the ones that are also associated with cancer. Some of the factors associated with dysbiosis and cancer are low socioeconomic status, ethnicity, poor access to medical care, a high prevalence of sexually transmitted diseases, smoking, alcohol consumption, obesity, reduced physical activity, metabolic syndrome, high levels of stress, aging, hormonal imbalances, genetic and epigenetic factors, impaired immunity, the human papillomavirus [22,23]. Smoking, douching, and obesity were all linked to bacterial vaginosis [2].

Changes in the microbiome also induce complex changes in human cells [24]. From a biological perspective, the normal cervicovaginal microbiome is composed mainly of *Lactobacillus* spp., thus exhibiting low bacterial diversity and protecting against carcinogenesis through various mechanisms [25]. The lactobacilli secrete lactic acid, and the low vaginal pH promotes healthy local homeostasis. The lactobacilli also secrete cytokines, antimicrobial peptides, and other metabolites that protect the local epithelium. They promote a healthy level of physiological inflammation that stimulates the immune system to fight against pathogens.

On the other hand, the dysbiotic cervicovaginal microbiome exhibits a high diversity of microorganisms, primarily obligate and strict anaerobes, that lead to a high vaginal pH. The bacteria promote the disruption of the epithelial barrier and secrete various metabolites and enzymes such as sialidase, proinflammatory cytokines and chemokines, reactive oxygen species, and other carcinogenic metabolites that lead to chronic inflammation and a dysregulated local metabolism. Further down the line, they also lead to genotoxicity and genomic instability, as well as altered proliferation and altered apoptosis. The dysbiotic environment also promotes angiogenesis. The chronic inflammation activates immune cells that secrete even more proinflammatory cytokines and chemokines such as Interleukin (IL)-6, IL-8 or Tumor necrosis factor (TNF), resulting in even more reactive oxygen species that further promote carcinogenic mechanisms. Hence, there are many different mechanisms through which the microbiota can impact carcinogenesis [25,26,27].

### 2.1. The Microbiome and Endometrial Cancer

Whereas the most common gynecological cancer in developing countries is cervical cancer, because of high rates of Human Papilloma Virus (HPV) infection and low rates of vaccination, the most common gynecological cancer in developed countries is endometrial cancer [2]. Many factors are associated with endometrial cancer, including high estrogen levels, obesity, chronic inflammation, and post-menopausal hormonal therapy.

The gut microbiome and the circulating estrogen levels are intensively connected as a feedback loop, influencing each other. We can hypothesize that the gut microbiome, the estrobolome in particular, has a part to play in the development of endometrial cancer, but more research is needed. Moreover, estrogen metabolism and the gut and vaginal microbiome are influenced by obesity. There is an association between the body mass index, the estrogen metabolism and the composition of the vaginal and gut microbiome [2,28].

A high vaginal pH is correlated with endometrial cancer, usually due to a disbalance of the vaginal flora. For instance, recent studies showed that *Atopobium vaginae* and *Porphyromonas* among other bacteria that raise the vaginal pH are more prevalent in the vaginal flora of women with endometrial hyperplasia or endometrial cancer [29]. It is believed that this promotes chronic endometrial inflammation that turns on the carcinogenesis process [2].

Compared with benign uterine lesions, endometrial cancer is associated with a decrease in the diversity of the local endometrial microbiota [30]. Some less-represented endometrial carcinoma species are *Salinibacter ruber, Bacillus tropicus, Pusillimonas* sp., *Riemerella anatipestifer*, *Nostocales cyanobacterium* HT-58-2 and *Corynebacterium pseudotuberculosis* [31]. This leads to an overgrowth of the remaining species. Micrococcus overgrowth is associated with an inflammatory profile in endometrial cancer, with increased IL-6 and IL-17 mRNA levels. *Bilophila, Rheinheimera*, *Rhodobacter*, *Vogesella* and *Megamonas* are overgrown in benign uterine lesions [30]. *Atopobium vaginae* and *Popayromonas somerae* induce the production of proinflammatory cytokines IL-1α, IL-1β, IL-17α, and TNFα; they also alter the transcription of CCL13, CCL8, CXCL2, IL22 and IL9 [32]. The production of IL-17α induces the production of IL-8 and TNFα, which are promoting factors for endometrial cell proliferation and angiogenesis [33]. TNFα also contributes to resistance to chemotherapy and metastasis development [34]. In endometrial cancer, IL1α and IL1β are overexpressed and promote cell proliferation, adhesion, invasion, and angiogenesis [35].

### 2.2. The Microbiome and Ovarian Cancer

Ovarian cancer is a relatively rare tumor with a bad prognosis since it develops inconspicuously with no symptoms until the late stages.

Genital dysbiosis has been associated with ovarian cancer, although more research is needed to draw causality conclusions [36]. Sexually transmitted bacteria such as *Chlamydia* spp. and *Mycoplasma* spp. that cause chronic reproductive tract inflammation have been associated with ovarian cancer. For instance, more than 60% of ovarian tumors contain such intracellular bacteria [2]. Other microorganisms associated with ovarian cancer are *Proteobacteria*, *Acinetobacter* spp., *Brucella* and even viruses such as cytomegalovirus or HPV [2,37,38].

Lactobacilli species in the cervicovaginal part of the genital tract have a protective role against ovarian cancer [39]. BRCA mutation carriers are associated with a reduction in *Lactobacillus* spp. This association is more substantial in younger patients [40].

An increase in *Proteobacteria* and *Fusobacteria* characterizes the microbiome in the tumor tissue compared to normal tissue; these gram-negative bacteria make the microbiome more immunogenic [41,42,43].

Pelvic inflammatory disease is a risk factor for ovarian cancer [2,44,45,46]. Bacterial flagellin and lipopolysaccharide (LPS) have an essential role in driving inflammation in ovarian cancer by inducing a response in pattern recognition receptors TLR2, 4, and 5 [41,47,48,49,50,51,52,53,54], leading to activation of NF-kappa B signaling [42]. LPS stimulate cancer cells inducing PI3K activation, EMT and overexpression of Vimentin, Snail, α-SMA, TCF, MMP2, N-cadherin, Slug, and MMP9 [53]. Even though LPS activates tumoral-associated macrophages, pushing them towards the M1 profile [55,56] and making them cytotoxic and cytostatic for ovarian cancer cells [57], a recent study has shown that administration of LPS does not prolong and may even shorten survival [58].

The increase in Gram-negative bacteria leads to an increase in lysophospholipids, which are by-products of bacterial metabolism [59,60]. Lysophosphatids are similar to lysophospholipids; in ovarian cancer patients, lysophosphatids plasma levels are increased [61,62]. In ovarian cancer cells, lysophosphatidic acid can increase the expression of angiogenesis promoters [63] and induce cell migration, invasion and proliferation [64,65,66,67,68,69,70]. A short description of bacterial metabolites effects on ovarian cancer is displayed in Table 1.

Bacteria metabolize tryptophan, producing indole-derivatives [71,72,73,74,75,76], which act on the aryl hydrocarbon and pregnane X receptors [77,78,79]. Aryl hydrocarbon receptor is involved in immune regulation [76,80]. Tryptophan rich diet leads to the proliferation of Lactobacilli [77], which prevents the proliferation of pathogenic bacteria [77,81,82,83]. Tryptophan and indolepropionic acid levels are reduced in the serum of ovarian cancer patients [84,85,86,87,88] and are inversely correlated with the stage of the disease [88].

Antibiotics (glycylcyclines, erythromycins, tetracyclines and chloramphenicol) can block cellular proliferation and reduce the proportion of ovarian stem cells [89]. Minocycline [90,91,92,93], Ciprofloxacin [94], and Salinomycin [87,95,96,97,98,99,100] can reduce the proliferation rate of ovarian cancer cells. In murine models, antibiotics can also be used to prevent cisplatin resistance [101], and minocycline can potentiate the activity of topoisomerase inhibitors [102].

Even though many studies suggest a potential benefit of antibiotic therapy, there is a study in which the treatment of mice grafted with ovarian cancer with neomycin, ampicillin, vancomycin, and metronidazole was associated with increased invasiveness and growth of the grafts [103].

**Table 1:** Effects of bacterial metabolites on ovarian cancer.

Bacterial Flagellin	Activation of NF-kappa B Signaling [42]
Lipopolysaccharides	PI3K activation, EMT, overexpression of Vimentin, Snail, α-SMA, TCF, MMP2, N-cadherin, Slug, and MMP9 activation tumoral-associated macrophages [53]
Lysophosphatids	angiogenesis, cell migration, invasion and proliferation [61,62]
Indole-derivatives	immune regulation [76,80]

### 2.3. The Microbiome and Cervical Cancer

Cervical cancer is a common malignancy in women, especially in developing countries where the HPV vaccination rate is low. Over 99% of cervical cancer biopsies contain HPV Deoxyribonucleic acid (DNA) as determined by Polymerase chain reaction (PCR) [2,104]. HPV is the major carcinogenic factor in the evolution of cervical cancer through the expression of E6 and E7 proteins. The most high-risk genotypes are HPV 16 and HPV 18. However, it is essential to note that 85–90% of HPV infections with high-risk genotypes are spontaneously cleared [2]. The high-risk HPV infections that persist can, in time, lead to cervical intraepithelial neoplasia (CIN)—low grade and then high grade—and then progress to invasive cervical cancer.

The link between vaginal dysbiosis and HPV persistence and neoplastic transformation is yet to be established. Still, various studies have already shown that the composition of the cervicovaginal flora differs in women with different HPV statuses [105,106]. HPV persistence has been linked with bacterial vaginosis by various studies, and anaerobic flora is conducive to HPV persistence [2,105,106,107]. A high vaginal bacterial diversity and a depletion of *Lactobacillus* spp. have been repeatedly associated with a low clearance of HPV.

HPV-negative women have been shown to host mainly *Lactobacillus crispatus* and *Lactobacillus iners*. However, HPV-positive women with a normal cervix contain the two lactobacillus species in different proportions. The risk of cervical transformation is higher with *Lactobacillus iners* than with *Lactobacillus crispatus* [108]. Once the HPV infection progresses toward cervical intraepithelial neoplasia, the cervicovaginal bacterial diversity increases correspondingly. The *Lactobacillus* spp is depleted, and the vaginal pH is elevated. The highest diversity is found in invasive cervical cancer (*Fusobacterium necrophorum*, *Gardnerella vaginalis*, *Sneathia* etc.) [2,108,109].

Various studies have shown that vaginal *Sneathia* associates with HPV persistence and pathological progression to cancer. *Atopobium* spp. is also associated with HPV persistence [110].

Other organisms that have been shown to influence the transformation of HPV lesions are *Candida albicans*, *Chlamydia trachomatis* and *Ureaplasma urealyticum* [2].

The increase in the diversity of the microbial flora leads to the production of cytokines which amplify the inflammatory response [108,111,112,113], leading to immune dysregulation in the reproductive tract and thus creating a more suitable site for tumor development [24].

*Mycoplasma genitalium* causes bacterial cervicitis and vaginitis, increasing the incidence of cervical lesions [114,115]. *Chlamydia trachomatis* damages the cervical mucosa and promotes infection of the cervical epithelium by HPV [116,117]. See Table 2 below.

Fusobacterium leads to increased production of interleukin-4, interleukin-10 and TGF- beta1 in the cervix and vagina; these cytokines are also increased in cervical cancer and squamous intraepithelial disease [118].

## 3. Interaction between Cancer Treatment and the Microbiome

The main pillars of cancer treatment are surgery, radiotherapy, chemotherapy, targeted molecules, and immunotherapy. This part of the article explores the interaction between cancer treatment and the microbiome. We will summarize what is known on the female reproductive tract microbiome, and in addition we will also explore the gut microbiome. The gut microbiome is much more investigated, and we hope that these insights will lead to new interesting research projects on the female reproductive tract microbiome as well.

Moreover, understanding the gut microbiome is important because a lack of oestrogen-metabolizing bacteria (from a lower diversity of the gut microbiota after chemotherapy for instance) could influence the vaginal microbiome composition. Therefore, strategies targeted towards the gut microbiome might have an indirect effect on the vaginal microbiome as well.

It is well-known that both radiotherapy and chemotherapy can cause gut mucositis and diarrhea. They also decrease the diversity of the gut microbiome, which is usually linked to digestive tract side effects. In contrast, radiotherapy and chemotherapy seem to increase bacterial diversity of the female reproductive tract, and increased bacterial diversity is a sign of disease, as previously explained.

Immunotherapy has emerged as a treatment in multiple types of cancer in recent years. Regarding gynecological cancers, it is of interest especially in patients with MSI-H endometrial, cervical, and ovarian cancer. Not much is yet known about the effects of immunotherapy such as Nivolumab, Ipilimumab and Pembrolizumab on microbiomes. However, we can hypothesize that there is an interesting interplay between immunotherapy and microbiomes since they both act on and modulate the immune system. More research is needed in this direction. 

Some specific bacteria-like microorganisms, such as *Bifidobacterium longum*, *Ruminococcaceae* and *Akkermansia muciniphila* were found to be more abundant in fecal samples collected from PD-1-responding patients. Oral supplementation with *Akkermansia muciniphila* proved beneficial in restoring response to immunotherapy in mouse models of epithelial tumors. The authors noticed an increase in the recruitment of CCR9+, CXCR3+, CD4+ T lymphocytes [119]. Proposed mechanisms involve the production of short-chain fatty acids and their pro-apoptotic role in cancer cells through activation of p21 cell cycle inhibitor and specific caspases, but also activation of the mTOR-S6K and STAT3 pathways in T-cells [120]. Administration of an oral cocktail of live Bifidobacterium to tumor-bearing mice significantly improved tumor control for several weeks. The same mice presented elevated levels of tumor-specific T cells in the periphery and antigen-specific CD8+ T cells within the tumor. Authors noticed a lack of anti-tumor effect in immunodeficient mice or mice treated with previously heat-inactivated Bifidobacterium [121]. Opposite results come from the study of Kim et al., who expanded on parabiotics as non-viable microbial cells in the form of heat-killed Bifidobacterium or Lactobacillus. These strains induced apoptosis of human colorectal carcinoma RKO cells in vitro and also revealed anti-tumor effects in an RKO cell-derived xenograft model through the activation of caspase-9, 3, 7 and PARP [122].

Interestingly, antibiotics seem to decrease immunotherapy’s efficacy, suggesting a link between these novel treatments and the microbiomes. Antibiotics also seem to increase the toxicity of chemotherapy. Moreover, radiotherapy, chemotherapy and immunotherapy are all less efficient in a germ-free mouse; fecal-matter transplantation and probiotics have been shown to improve the efficacy of immunotherapy [2,123,124,125,126].

The gut microbiota may be involved in the prevention of chemotherapy-associated toxicity, improved efficacy of oncologic treatment, prevention of surgical morbidity, and quality of life. Diarrhea, abdominal pain, vomiting, and weight loss are critical adverse reactions to chemotherapy that cause significant morbidity. Preventive intervention on the gut microbiota can influence the pathogenesis of mucositis through TLR2 signaling, mediation of vitamin B production, and microbial enzymatic degradation. Additionally, prognostic markers can be derived from specific microbiota patterns. The bowel mucosa load with *Fusobacterium nucleatum* strains correlates with worse prognostic in patients with colorectal cancer [125].

Modulating microbiomes had essential health benefits in many chronic and inflammatory diseases, including irritable bowel syndrome and recurring *Clostridioides difficile* infections and implications in cancer prevention and response to treatment.

Gut microbiota modulation is represented by probiotics, prebiotics, antibiotics or other drugs, or microbiota transplantation [127].

Bifidobacterium and Bacteroides species have been associated with immune modulation and estrogen metabolism and are under investigation for preventing estrogen-derived cancer such as breast, endometrial, and ovarian cancer [2]. Probiotics containing *Lactobacillus lactis* engineered to secrete an antimicrobial peptide involved in gut homeostasis (pancreatitis-associated protein) proved to reduce enteritis induced by 5-Fluorouracil in cancer patients. The mechanism was represented by a reduced abundance of pathogenic bacteria such as *Enterobacteriaceae* in the intestine, thus reducing the intensity of mucositis [128]

Fecal microbiota transplantation reduced the side effects generated by chemotherapy and radiotherapy [129]. However, the most important studies are related to fecal microbiota transplantation from responders to germ-free mice with xenograft tumors (melanoma, lung or kidney) which showed an increased response to checkpoint inhibitors [130].

Approaches for modulating vaginal microbiomes are under investigation. They aim to modify vaginal microbiota to optimal Lactobacillus-dominant flora to prevent carcinogenesis and in cancer patients to increase the effectiveness of treatments and decrease toxicity. Novel antimicrobials and probiotics such as intravaginally delivered vaginal lactobacilli formulations, biofilm disruptors, and vaginal microbiota transplantation are being considered.

Vaginal probiotic lactobacilli (*L. crispatus* strain CTV-05 known as LACTIN- V) have been tested with success in clinical trials, mainly for the treatment of bacterial vaginosis or urinary tract infection (UTI) [131,132].

Vaginal microbiota transplantation (VMT) from donors with optimal vaginal flora is a novel potential treatment option under investigation for women with vaginal disorders. However, there is an unknown long-term risk of microbiome transplants (fecal or vaginal) related to the potential transfer of antimicrobial-resistant microorganisms, which may be problematic in immunodepleted cancer patients.

Probiotics consisting of *Lactobacillus* spp. might aid in the treatment of cervicovaginal dysbiosis and persistent HPV infections [133,134]. *Lactobacillus* spp. probiotics might increase the clearance of HPV when used long-term in certain patients [134,135]. Since it is well established that persistent HPV infections increase the risk of cervical cancer, *Lactobacillus* spp. probiotics might be considered in HPV positive patients. However, more research is needed before establishing clear links and then guidelines.

A study conducted by Tsementzi et al. showed that radiation therapy alone in post-menopausal patients with gynaecologic cancer leads to a perturbation of the vaginal microbiome with a decrease of *Lactobacillus* spp. The study showed a higher vaginal bacterial diversity in cancer patients with respect to healthy patients and a higher vaginal bacterial diversity in post-radiotherapy with respect to pre-radiotherapy. This might be associated with some post-radiotherapy symptoms in patients with vulvovaginal atrophy and these findings might have implications for future therapeutic interventions, such as probiotics or vaginal microbiome transplantation [136].

Overall, not much is known about the female reproductive tract microbiome and its changes during cancer treatment, and even less is known on the influence of the female reproductive microbiome on the response to various treatments.

## 4. The Microbiome and Endometriosis

Endometriosis is a multifactorial disease whose etiology is not entirely established. One theory is “retrograde menstruation” where the menstrual flux and viable endometrial cells go through the fallopian tubes to the peritoneum, where they adhere. There is an essential component of inflammation, but it is not yet clear if this is a cause or an effect of endometriosis. Interestingly, the composition of the gut microbiome is also linked to this disease. A healthy gut is composed of a balanced distribution of *Firmicutes* spp. and *Bacteroidetes* spp. However, in endometriosis, this balance is altered with a predominance of either one or the other species. Endometriosis development can induce a change in the gut microbiome [137,138]. The complex interrelation between endometriosis, circulating estrogen levels, and gut bacteria warrants further research.

## 5. Conclusions

The microbiome, in general, and the female reproductive tract microbiome, is an exciting research avenue. More and more studies show a connection between different microbiome compositions and various cancers. There is a low diversity of bacterial species in the vagina and cervix, represented mainly by *Lactobacillus* spp. which prevents colonization of the female genital tract with pathogenic bacteria. The proliferation of pathogenic bacteria leads to a higher diversity of the microbiome. This abnormally diverse microbiome can modulate the immune response in the female genital tract creating an environment characterized by chronic inflammation, which is favorable for developing neoplasia. Some products of bacterial metabolism have carcinogenic properties and act upon the normal cells of the genital tract leading to genetic alterations. Other products of bacterial metabolism have angiogenic properties and promote neovascularization, which favors vascular invasion and metastasis.

Moreover, the microbiome also seems to influence the response to therapy and toxicity. The estrobolome, through its effect on estrogen circulating levels, can impact both the composition of the cervicovaginal microbiome and carcinogenesis. More research is needed to describe these interactions better and find ways of harnessing this information toward better treatments.

## Figures and Tables

**Table 2:** Examples of bacteria associated with female reproductive tract pathology.

**Bacteria found to be associated with female reproductive tract pathology, including cancer**	*Popayromonar somerae*, *Chlamydia* spp., *Mycoplasma* spp., Proteobacteria, *Acinetobacter* spp., *Brucella* spp., *Fusobacterium necrophorum*, *Gardnerella vaginalis*, *Sneathia* spp., *Candida albicans*, *Chlamydia trachomatis*, *Ureaplasma urealyticum*	
**Examples of how bacteria might induce pathologic changes in the female reproductive tract**	*Mycoplasma genitalium*	Cervicitis and vaginitis, chromosomal lesions [114,115]
	*Chlamydia* *trachomatis*	Increased risk of infection of the cervical epithelium by HPV [116,117]
	*Fusobacterium* spp.	Increased production of IL-4, IL-10 and TGF-b1 [118]
